# Utilization and cost of a new model of care for managing acute knee injuries: the Calgary acute knee injury clinic

**DOI:** 10.1186/1472-6963-12-445

**Published:** 2012-12-05

**Authors:** Breda HF Lau, Mark R Lafave, Nicholas G Mohtadi, Dale J Butterwick

**Affiliations:** 1 Department of Physical Education and Recreational Studies, Mount Royal University, 4825 Mount Royal Gate SW, Calgary, AB T3E 6K6, Canada; 2 Department of Medical Science, Faculty of Kinesiology, University of Calgary, Calgary, AB, Canada; 3 Faculty of Kinesiology, University of Calgary, Calgary, AB, Canada

## Abstract

**Background:**

Musculoskeletal disorders (MSDs) affect a large proportion of the Canadian population and present a huge problem that continues to strain primary healthcare resources. Currently, the Canadian healthcare system depicts a clinical care pathway for MSDs that is inefficient and ineffective. Therefore, a new inter-disciplinary team-based model of care for managing acute knee injuries was developed in Calgary, Alberta, Canada: the Calgary Acute Knee Injury Clinic (C-AKIC). The goal of this paper is to evaluate and report on the appropriateness, efficiency, and effectiveness of the C-AKIC through healthcare utilization and costs associated with acute knee injuries.

**Methods:**

This quasi-experimental study measured and evaluated cost and utilization associated with specific healthcare services for patients presenting with acute knee injuries. The goal was to compare patients receiving care from two clinical care pathways: the existing pathway (i.e. comparison group) and a new model, the C-AKIC (i.e. experimental group). This was accomplished through the use of a Healthcare Access and Patient Satisfaction Questionnaire (HAPSQ).

**Results:**

Data from 138 questionnaires were analyzed in the experimental group and 136 in the comparison group. A post-hoc analysis determined that both groups were statistically similar in socio-demographic characteristics. With respect to utilization, patients receiving care through the C-AKIC used significantly less resources. Overall, patients receiving care through the C-AKIC incurred 37% of the cost of patients with knee injuries in the comparison group and significantly incurred less costs when compared to the comparison group. The total aggregate average cost for the C-AKIC group was $2,549.59 compared to $6,954.33 for the comparison group (p <.001).

**Conclusions:**

The Calgary Acute Knee Injury Clinic was able to manage and treat knee injured patients for less cost than the existing state of healthcare delivery. The combined results from this study show that the C-AKIC is an appropriate, effective, and efficient model of clinical care for patients presenting with acute knee injuries.

## Background

Timely access to healthcare is dependent upon obtaining health services in the most suitable setting, in a reasonable time, and within a reasonable distance [1]. One area in need of improved patient flow and integrated services is the management and treatment of musculoskeletal disorders (MSDs). MSDs affect a large proportion of the Canadian population and are a leading cause of illness and disability with 15.5 million visits made to ambulatory physicians annually [2]. Nearly 45% of visits to primary care physicians are related to MSDs, however only 8% of primary care services are dedicated to MSD management [3,4]. Furthermore, MSDs present a huge problem that will continue to strain primary care resources [5].

Currently, the literature has suggested that the clinical care pathway for MSDs in Canada is inefficient whereby patients do not often receive best practice treatment [6-9]. One issue stems from a lack of clinical confidence in primary care examination of MSDs as well as “a lack of cognitive mastery in basic musculoskeletal medicine” [4]. Recent studies have suggested that there is a relationship between MSD mishandling and physician ineffectiveness in management of MSDs which likely stems from educational deficiencies at the medical school level [10-16]. Primary care training programs often do not include specific experience with MSDs [17] resulting in a lack in basic education of MSD and coordination between different disciplines.

A second issue that affects the ability to provide best practice MSD treatment and management stems from a shortage of physicians, specialists, facilities, hospital beds, and medical equipment in Canada [18-20]. In 2003, more than 1.2 million Canadians aged 15 and over were unable to find a family physician because only 1 in 12 family physicians were accepting new patients [21]. In 2001, 1 in 10 Canadians saw a healthcare specialist each year, yet there was only 1 specialist for every 1,077 people [18,22]. The literature suggests that short-term solutions aimed at increasing the supply of physicians will not translate into improvements where the need for care is most critical [18]. New models of healthcare delivery must be designed and practitioner roles must be reshaped. A call has been issued for cooperation among governments, healthcare providers, patients, and strategic investments. The goal is to shift away from traditional paradigms in order to seize new opportunities and take advantage of new approaches [19].

Therefore, a new inter-disciplinary team-based model of care for managing acute knee injuries was developed in Calgary, Alberta, Canada: the Calgary Acute Knee Injury Clinic (C-AKIC) [23]. The C-AKIC employs both primary care physicians and a new healthcare practitioner, the “Non-Physician Expert” (NPE). The NPE is an individual with a background in MSDs, such as a certified athletic therapist, who is trained at a specialist’s competency level in an interdisciplinary team with a supervising physician [23]. In addition, the C-AKIC utilizes a new portal of entry into the healthcare system through self-referral and a unique web-based screening and diagnostic process.

The overall goal of the C-AKIC project was to employ a quality-focused clinical care pathway for the management and treatment of knee injuries. The literature suggests that a quality-focused healthcare system will be capable of preventing and managing illness, which will ultimately decrease healthcare costs and improve overall quality of care [1]. A Healthcare Access and Patient Satisfaction Questionnaire (HAPSQ) was utilized to collect data that could measure and evaluate the quality of the C-AKIC. Quality was defined using an evaluative framework called the Alberta Quality Matrix for Health (AQMH), which was developed by the Health Quality Council of Alberta for measuring health service quality using six service quality areas: accessibility, efficiency, acceptability, effectiveness, safety and appropriateness [1]. The AQMH was used as an evaluation guide and HAPSQ data were mapped according to the six quality indicators. Mohtadi et al., 2012 evaluated and reported on the acceptability, accessibility, appropriateness, effectiveness, efficiency, and safety of the C-AKIC in terms of access to care, wait times, and patient satisfaction [23]. The goal of this paper is to evaluate and report on the appropriateness, efficiency, and effectiveness of the C-AKIC through healthcare utilization and costs associated with acute knee injuries.

## Methods

### Design and study population

The purpose of this study was to evaluate utilization and costs associated with acute knee injuries through two clinical care pathways: the existing pathway and a new model, the C-AKIC. This was accomplished through the use of the HAPSQ. The HAPSQ is a tool that was designed to measure access and patient satisfaction (as identified in Mohtadi et al., 2012) but it was also designed to measure costs associated with acute knee injuries. To be eligible for inclusion, patients within the ages of 14 and 65 had to present with acute injuries within 3 months from the onset of symptoms (e.g., pain, swelling, instability) occurring from either an overuse or traumatic mechanism. Selection criteria also included patients presenting with the following diagnoses: locked knees, meniscal tears, and/or ligament tears. Patients with knee injuries treated on an inpatient basis (e.g., dislocated knees, fractures, infections) or suffering from language, cognitive, and psychiatric difficulties were excluded. Ethics approval for the study was provided by the Conjoint Health Research Ethics Board (CHREB) at the University of Calgary.

The research design involved a quasi-experimental study conducted between May and December 2008 in which the HAPSQ was distributed to two groups of patients: (a) an alternative pathway experimental group (i.e., the C-AKIC group) and (b) a comparison group representing the current state. The experimental group was a convenience sample of patients who received the alternate pathway of care through the C-AKIC. All patients receiving care through the C-AKIC between May and December 2008 were invited to complete the web-based HAPSQ. The comparison group (i.e. current state) was represented by a convenience sample of patients receiving the existing standard of care for their knee injuries in Calgary (and region), Alberta, Canada geographical region. Patients eligible for the study were recruited by a research assistant during scheduled physician appointments at the University of Calgary Sport Medicine Centre (U of C SMC) and Banff Sport Medicine Clinic. In an effort to obtain a representative sample from across Alberta, poster advertisements were sent to all primary care physician clinics across Alberta. Patients recruited using this method were asked to contact a research assistant for screening and instruction. Patients in both groups were asked to provide written informed consent prior to receiving the questionnaire.

### Estimation of resource utilization and costs

The outcomes of interest were utilization and costs of total and specific categories of health services. Questionnaire responses were based on patient information recorded from the date of injury until definitive treatment was received and the physician discharged the patient. Socio-demographic information included age, gender, ethnicity, marital status, working status, and income. Cost information included use of healthcare resources, use of over-the-counter (OTC) and prescription medications, out of pocket expenses, and economic loss of missed work. Cost estimates were divided into three categories: 1) costs to the patient; 2) costs to the province of Alberta; and 3) costs to private insurance companies. When possible, information from detailed costing data from reliable databases such as billing records, medical benefits’ and pharmaceutical price lists [24,25] and government costing reports [26] were used to estimate costs. All cost estimates were calculated in Canadian dollars (CAD$). Cost transformations were not adjusted for annual deflation and inflation factors during the 2008 time horizon.

#### Costs to the patient

Costs to the patient were defined as the total cost of out-of pocket expenses incurred by the patient including lost wages. Lost wages were quantified in number of days taken off work multiplied by hourly occupational wage estimates obtained through the Alberta Bone and Joint Health Institute’s detailed costing database. Variables used to estimate out-of-pocket expenses included private diagnostic services, over-the-counter medication, healthcare appliances and aids (e.g., canes, toilet seats), accommodations, travel/transportation, and parking. Patients were asked to self-report quantity of units purchased and costs were estimated using a variety of means. The average cost for out-of-pocket MRI and CT scan costs were estimated by contacting private diagnostic facilities across the province. Estimates for OTC medications were calculated using drug names, size of the bottle, and number of bottles reportedly purchased by each patient (e.g., Advil, 400 mg or 400 mL or Extra Strength, 2 bottles). OTC medication costs were priced using the Alberta Health and Wellness’s Interactive Drug Benefit List database, which is an online, searchable database for Alberta Government-sponsored drug programs and includes a comprehensive manufacturer’s price listing for essential prescription drugs, non-prescription drugs, and nutritional products [24]. Pharmaceutical costs were estimated based on actual expenditures (i.e. how many pills were actually consumed). OTC supports, appliances, and braces were estimated using average retail prices for appliances obtained from various medical supply stores, pharmacies, and retailers. Total cost estimates for accommodation, travel/transportation, and parking were self-reported.

#### Costs to the province

Costs to the province were defined as the total costs associated with diagnostic tests rendered, ambulatory visits, surgery, and physician visits. Physician visits were further subdivided into primary (e.g., general practitioner, family physician) and ‘specialty’ care (e.g., sport medicine physician, orthopaedic surgeon) visits using provider codes. Patients were asked to self-report quantity of units utilized and type of services rendered. Cost estimates for diagnostic procedures and services, out-patient ambulatory care, and in-patient surgical interventions were obtained from the Alberta Health and Wellness Health Costing in Alberta Annual 2006 [26]. Cost valuations for physician visits were based on billing codes from the Alberta Health Care Insurance Plan Medical Benefits Price List (April 2009) [25].

#### Costs to insurance companies

Costs to insurance companies were defined as costs that were covered by third party insurance (i.e., not provincial insurance). Variables used to estimate third-party insurance costs included prescription medication, therapeutic or rehabilitative treatments and services, and custom-made supports, appliances, and braces. Patients were asked to self-report quantity of units purchased and costs were estimated using a variety of means. Cost valuations for prescription medications were calculated using drug names and multiplying the number of tablets prescribed with price per tablet listed in Alberta Health and Wellness’s Interactive Drug Benefit List database [24]. Pharmaceutical costs were estimated based on actual expenditures and not pill consumption. Cost estimates for therapeutic or rehabilitative treatments and services were based on billing guidelines and/or fee schedules provided by provincial and national governing bodies for each profession (e.g., chiropractors, physiotherapists). Average cost estimates for custom-made supports, appliances, and braces were estimated using retail prices for appliances obtained from various medical supply and orthotic companies across the province.

### Data analysis

Data was analyzed using SPSS©, version 17.0. Independent t-tests were employed to determine if there were statistical utilization and cost differences between the experimental group and the comparison group. Post-hoc analysis of the comparison group to the intervention group was completed to ensure there were no differences between groups for sex, marital status and income using Chi-Squared test for independence and an independent t-test for age. Pearson’s correlations were used to assess the relationship between total costs with socio-demographic data and clinical visits to a variety of healthcare providers. A P value of less than .05 was considered statistically significant for all analyses.

## Results

In the experimental group, 274 patients were consented from which 139 patients returned a HAPSQ, resulting in a response rate of 50.4%. One questionnaire was missing data. Therefore, data was analyzed from 138 (50.3%) questionnaires. In the comparison group, 275 patients were approached to participate in the study, in which 168 (61.1%) HAPSQs were returned. Thirty-two questionnaires were missing data resulting in the analysis of 136 (49.5%) questionnaires. For both groups, the main reasons for nonparticipation were time and effort. Patient characteristics comparing both groups are shown in Table 1. The experimental and comparison groups were statistically similar sex [χ^2^(1) = .53, *p* = .47], race [χ^2^(1) = 4.69, *p* = .46], marital status [χ^2^(1) = 8.57, *p* = .13], income [χ^2^(1) = 4.32, *p* = .63], and age [*t*(272) = .48, *p* = .49] in all respects.

**Table 1:** Characteristics of the study sample

		**C-AKIC (Experimental) (n=138)**		**Current State (Comparison) (n=136)**	
**Age** (**years**)	Mean (SD)*	39.2	(14.3)	33.6	(13.2)
	Range	11-73		14-65	
**Male**: **n** (%)		70	(50.7)	62	(45.6)
**White**: **n** (%)		126	(90.6)	116	(85.3)
**Marital Status**: **n** (%)	Married	68	(49.3)	50	(36.8)
	Living with Partner	11	(7.9)	14	(10.3)
	Other	59	(42.8)	72	(52.9)
**Income**: **n** (%)	> $60 000	29	(21.0)	39	(28.7)
	$ 60 000 – 80 000	10	(7.2)	16	(11.8)
	$ 80 000 – 100 000	19	(13.8)	15	(11.0)
	> $100 000	44	(31.9)	35	(25.7)
	Prefer not to say	36	(26.1)	31	(22.8)

*SD: Standard deviation.

C-AKIC: Calgary Acute Knee Injury Clinic.

### Healthcare utilization and costs

A summary of provincial healthcare services utilization are presented in Table 2. Patients receiving care for their acute knee injury used less provincial healthcare services than patients receiving care through the existing clinical care pathway. Patients receiving care through the C-AKIC made 0.61 times the amount of primary care physician (1.01 vs 1.66) and 0.68 specialist visits (2.53 vs 3.72) compared to the comparison group. C-AKIC patients also made 0.31 times the number of visits to the emergency department (0.17 vs 0.54). With respect to diagnostic imaging, MRIs were ordered by practitioners in the C-AKIC 0.32 (0.21 vs 0.65) times the number of MRIs in the comparison group. The utilization differences between the two groups of patients was statistically significant (p < .005) for all provincial healthcare services (Table 2).

**Table 2:** Use of provincial healthcare services by patients with acute knee injuries

**Cost Variable**	**Experimental Group (C-AKIC) n = 138**		**Comparison Group (Current State) n = 136**		*p *Value*	**95% Confidence Interval**	Utilization Ratio**
	**Mean**	**SD**	**Mean**	**SD**			
Primary care physician^+^ clinical visits	1.01	1.82	1.66	1.99	0.005	0.20-1.11	0.61
Specialist^++^ care clinical visits	2.53	1.26	3.72	3.03	<0.001	0.64-1.74	0.68
Emergency room visits	0.17	0.37	0.54	0.85	<0.001	0.18-0.50	0.31
MRI	0.21	0.40	0.65	0.54	<0.001	0.33-0.56	0.32

*t-test for independent samples.

**Utilization ratio = ratio of utilization for experimental group patients to utilization for comparison group.

^+^Primary care physician: family physicians and general practitioner.

^++^Specialist: sport medicine physicians, orthopaedic surgeons.

C-AKIC: Calgary Acute Knee Injury Clinic.

A summary of cost differences between groups is presented in Table 3. All cost variables between the two groups were statistically significant (p < .005). The total aggregate average cost for the C-AKIC group was $2,549.59 compared to $6,954.33 for the comparison group (*p* <.001). Overall, patients receiving care through the C-AKIC incurred 37% of the cost of patients with knee injuries in the current state. For specific categories, patients in the experimental group had 0.35 times the costs to the patient ($647.51 vs $1,855.64), 0.34 times the costs to provincial healthcare care ($1,205.32 vs $3,595.02), and 0.46 times the costs to insurance companies ($687.24 vs $1,503.67) when compared to patients in the comparison group. The MRI costs were taken out of the total provincial healthcare costs to investigate deeper into the contributing factors in the provincial healthcare costing variable. The experimental group spent 0.33 times ($109.32 vs $331.88) the costs of the comparison group resulting in a cost difference that was significantly different (*p* <.001).

**Table 3:** The cost differences for patients in the experimental group and the comparison group

**Cost Variable**	**Experimental Group (C-AKIC) n = 138**		**Comparison Group (Current State) n = 136**		*p *Value*	**95% Confidence Interval**	Cost Ratio**
	**Mean**	**SD**	**Mean**	**SD**			
Patient	647.51	1825.54	1855.64	4615.32	<0.005	375.54 -2040.73	0.35
Provincial healthcare	1205.32	1757.52	3595.02	2042.31	<0.001	1934.82 -2844.57	0.34
Insurance	687.24	795.17	1503.67	1247.79	<0.001	566.64 -1066.22	0.46
MRI	109.32	215.50	331.88	288.59	<0.001	161.75 -283.36	0.33
Total	2549.59	3172.73	6954.33	5953.35	<0.001	3265.86 -5543.62	0.37

*t-test for independent samples.

**Cost ratio = ratio of costs for experimental group to costs for comparison group.

Bivariate correlations between total costs with socio-demographic characteristics and clinical visits to different healthcare providers were performed (Table 4). There was a significant correlation between total cost and specialist (r = 0.361; p<.001) and emergency room visits (r = 0. 156; p<.001). There was not a significant relationship between total cost and visits to primary care physicians (r = 0.093; p=0.126). Age was the only socio-demographic variable significantly correlated to total cost (r = −0.301; p<.001).

**Table 4:** **Correlations between total costs with patients**’ **variables and visits to different healthcare practitioners**

**Variable**	Total Costs*	***p *Value**
Sex	−0.117	0.054
Race	0.093	0.126
Marital Status	−0.107	0.078
Income	0.005	0.930
Age	−**0.301**	<**0.001**
Primary care	0.093	0.126
Specialist	**0.361**	<**0.001**
ER	**0.254**	<**0.001**

*Pearson’s correlation; significant values in bold.

## Discussion

The overall goal of the C-AKIC project was to employ a quality-focused clinical care pathway for the management and treatment of knee injuries. The Alberta Quality Matrix for Health (AQMH) was used as a framework for evaluating six service quality areas: accessibility, efficiency, acceptability, effectiveness, safety, and appropriateness [1]. Mohtadi et al., 2012 evaluated and demonstrated acceptability, accessibility, appropriateness, effectiveness, and efficiency of the C-AKIC in terms of access to care, wait times, and patient satisfaction [23]. The study reported a significant difference in mean wait time from injury to physician discharge between the existing clinical care pathway (M=7.24 months, SD=6.75) and the C-AKIC (M=2.09 months, SD=1.86). Results also demonstrated a significant difference in patient satisfaction (M=89.43, SD=14.60 vs M=91.20, SD=13.25; *p*<.001) in terms of quality of care between primary care physicians in the existing clinical care pathway and C-AKIC specialists (i.e., NPEs).

This study evaluated the efficiency, effectiveness, and safety of the C-AKIC through healthcare utilization and its associated costs for patients presenting with acute knee injuries and receiving care from two different clinical care pathways. This was achieved by:

1) Measuring healthcare utilization and cost for patients receiving care through a new evidence-based model for management of acute knee injuries in Calgary, Alberta, Canada: the Calgary Acute Knee Injury Clinic (C-AKIC) model;

2) Measuring healthcare utilization and cost for patients receiving care through the existing clinical care pathway;

3) Determining the distribution of utilization across provincial cost variables;

4) Comparing total utilization and cost of patients receiving care for acute knee injuries in both pathways.

The results from this study show that utilization of provincial healthcare services (i.e., physician visits and MRI) was significantly greater in the comparison group (i.e. current state) over the experimental group (i.e. C-AKIC). Three different cost estimates were measured and compared: 1) costs to the patient; 2) costs to the province of Alberta; and 3) costs to private insurance companies. All cost variables were significantly more expensive in the comparison versus C-AKIC group. The combined results from both studies are in agreement with other studies that have examined the cost of waiting longer than medically recommended for treatment and found that longer wait times impose costs not only for the patients themselves, but also the economy as a whole [27]. Furthermore, there is a cost associated with loss of productivity and quality of life by patients while waiting for treatment which was not measured in this study. In 2011, as reported by the Fraser Institute (an independent Canadian non-partisan research and educational organization), there was an estimated 941,321 Canadians waiting for treatment and endured an estimated private cost of at least $1.08 billion [28]. This is in addition to the physical and psychological pain and suffering that may accompany these patients.

In addition to costs associated with lengthy wait times, delayed management and treatment of MSDs can result in the development of chronic disease and morbidity [29]. In particular, MSDs affecting the lower limb, particularly knee conditions, have consequences to the economy, healthcare system, and industry in terms of medical treatment, hospitalization, and lost working time [30]. Studies have strongly associated major joint injuries including cruciate ligament damage and meniscal tears with the onset of osteoarthritis [31]. Therefore early intervention, management, and treatment are important. Canadians, however, continue to report difficulties in accessing diagnostic tests, getting specialist appointments, accessing specialized services, and receiving non-emergency surgery [20,32,33]. Wait times for diagnostic tests are a major bottleneck to accessing other services. Results taken from the Alberta Waitlist Registry, 90% of Alberta’s patient population receiving MRIs average wait times between 4 to 6 months [34,35]. Although MRIs have been shown to be an accurate and effective tool for diagnosing ligamentous injuries [36,37] and meniscal tears [38,39], it is likely that a proportion of MRIs are being ordered inappropriately and some centres lack an effort to control demand by identifying inappropriate requests for MRI scans [40]. For instance, one study performed a chart audit on 666 patients presenting with acute knee injuries and found that of 142 MRIs were performed, only 35% of the cases met consensus-based MRI indications for appropriate MRI utilization [41]. Therefore, time is wasted while waiting for an expensive diagnostic test that potentially might not have been required in the first place. Furthermore, MRI resource utilization is being wasted and costs are increased.

Wait times for specialist services such as knee arthroscopy also serve as another bottleneck to accessing other services and also share a wait time between 4 to 6 months [34,35]. Evidence indicates that increasing resources will not result in the overall shortening of wait times because long term effects will experience increases in patient numbers [42]. Innovative policy directions are needed to guide the evolution of the health system resulting in a shift away from traditional paradigms in order to seize new opportunities and take advantages of new approaches [19]. This includes broadening the current scope of practice for allied healthcare professionals.

The current primary clinical care pathway utilizes too many practitioners on the primary care level because primary care physicians lack a basic education of MSD and a lack of coordination between different disciplines [6]. The literature shows that primary care physicians lack the necessary training [13] and confidence in musculoskeletal examination [43-46], which results in variable and inconsistent standards of care for knee injuries [47]. Therefore, wait times are increased because of variability with respect to how problems are diagnosed and managed, fragmentation of providers, and decreasing availability of healthcare practitioners making referrals to the appropriate provider at the right time [47]. Delays in treatment impact society and the healthcare system in terms of economic costs and quality of care.

Results evaluating the C-AKIC model of care demonstrated a unique and efficient approach to managing acute knee injuries in an urban setting. The C-AKIC model increased capacity and addressed staffing shortages without compromising the traditional roles of physiotherapists and nurses by utilizing the educational background, skill-set, and expertise of an athletic therapist. When comparing utilization for all provincial healthcare services, there was significantly less resource usage from patients receiving care through the C-AKIC. The C-AKIC employs innovative web-based screening technology whereby the patient is able to self-refer into the clinic. The patient is then contacted by an NPE in a timely fashion for an appointment. In the C-AKIC, a patient receives higher quality care because the NPE is able to deliver specialist care within an interdisciplinary team at a primary care level. The literature suggests that best practice MSD treatment outcomes for patients arise from a musculoskeletal problem that is assessed and managed by those with an appropriate level of expertise [6]. When the primary care physician is teamed up with a non-physician expert, the results are overwhelmingly positive from both a patient perspective [23] and from a leader or policy-maker perspective.

However, several limitations of this study must be pointed out. First, cost analysis was not calculated using direct cost measurement but on patient self-reporting of resource utilization on the HAPSQ. Although recall bias is often associated with self-report measures, studies have shown that resource utilization declines over time whereby patients have a tendency to under-report values resulting in a net underestimation of cost and utilization values [48,49]. The study also attempted to account for potential error by using conservative estimates of costs, and when possible, using detailed costing data from reliable databases such as billing records, medical benefits’ and pharmaceutical price lists [24,25], government costing reports [26].

Secondly, patients were recruited using a convenience sample, which may limit the extent to which the sample resembles the population of interest. Thus, selection bias is introduced and the generalizability of the findings may be compromized. Hultsch et al., 2002, however, suggests that samples drawn using random sampling procedures are not always representative of the population, and structured samples of convenience may yield results similar to those obtained from samples using random sampling strategies [50]. Therefore, all efforts were made to include patients from across the city of Calgary and province of Alberta. Furthermore, a post-hoc analysis of the comparison group to the intervention group was completed to ensure there were no significant differences between socio-demographic variables.

Modest response rates presented as another limitation in this study with respect to non-response bias. The response rates for the experimental and comparison groups were 50.4% and 61.1% respectively. This might have further introduced selection bias to the findings, however, there are currently no gold standards for what acceptable response rates should be. Though these rates were lower than anticipated, they were on par with recently published research that have suggested average trends for response rates of 53.8% for healthcare surveys and 54.7% for email surveys [51].

Finally, cost transformations were not adjusted for annual deflation and inflation factors during the 2008 time horizon, therefore, cost estimates were potentially under-estimated.

Despite these limitations, this study has important implications for MSD primary care. MSDs are the second most costly illness in Canada. [5,52] and have an economic burden of $17 billion per year and account for 39% of long-term disability [52]. A report released by the Conference Board of Canada, Canada’s Public Health Care System Through to 2020, Challenging Provincial and Territorial Capacity, indicates that projected health care expenditures are projected to reach 44% in 2020 from 32% in 2001 [53]. The significant burden of MSDs on the Canadian healthcare system emphasizes the need for improved patient flow and integrated services throughout the pathway of care. The C-AKIC was able to demonstrate a more cost-effectiveness, efficiency, and safe of model of clinical care. We are aware that the results may not be generalizable to the population sampled. However, healthcare is regionalized in many countries and lessons can be learned by these regions from the descriptors and variables of this study.

## Conclusion

The Calgary Acute Knee Injury Clinic was able to manage and treat knee injured patients for less cost than the current state of healthcare delivery. The combined results from this study and previous evaluation show that the C-AKIC is an acceptable, accessible, appropriate, effective, efficient, and safe model of clinical care for patients presenting with acute knee injuries.

## Competing interests

The authors declare that they have no competing interests.

## Authors’ contributions

BL, ML, and NM developed the study design. BL and ML carried out the statistical analysis. BL drafted the manuscript. NM and DB were involved in the conception of the study. All authors (BL, ML, NM, and DB) contributed to critically appraising, reviewing, and approving each draft of the manuscript. All authors read and approved the final manuscript.

## Pre-publication history

The pre-publication history for this paper can be accessed here:

http://www.biomedcentral.com/1472-6963/12/445/prepub
